# In Vitro Antitumor Activity of Aloperine on Human Thyroid Cancer Cells through Caspase-Dependent Apoptosis

**DOI:** 10.3390/ijms19010312

**Published:** 2018-01-21

**Authors:** Ying-Ray Lee, Shu-Hsin Chen, Ching-Yen Lin, Wen-Ying Chao, Yun-Ping Lim, Hui-I Yu, Chieh-Hsiang Lu

**Affiliations:** 1 Department of Medical Research, Ditmanson Medical Foundation Chiayi Christian Hospital, Chiayi 600, Taiwan; yingray.lee@gmail.com (Y.-R.L.); cych10472@gmail.com (S.-H.C.); jouyuan22@gmail.com (C.-Y.L.); 2 Department of Nursing, Min-Hwei College of Health Care Management, Tainan 736, Taiwan; april@mail.mhchcm.edu.tw; 3 Department of Pharmacy, College of Pharmacy, China Medical University, Taichung 404, Taiwan; limyp@mail.cmu.edu.tw; 4 Division of Endocrinology and Metabolism, Department of Internal Medicine, Ditmanson Medical Foundation Chiayi Christian Hospital, Chiayi 600, Taiwan; 04490@cych.org.tw; 5 Department of Biotechnology, Asia University, Taichung 404, Taiwan

**Keywords:** aloperine, thyroid cancer, apoptosis

## Abstract

The global incidence of thyroid cancer, one of the most common endocrine malignancies, is especially high among women. Although most patients with thyroid cancers exhibit a good prognosis with standard treatment, there are no effective therapies for patients with anaplastic thyroid cancers or cancers that have reached an advanced or recurrent level. Therefore, it is important to develop highly effective compounds for treating such patients. Aloperine, a natural compound isolated from *Sophora alopecuroides*, has been reported to possess antioxidant, anti-inflammatory, anti-neuronal injury, anti-renal injury, antitumor, anti-allergic, and antiviral properties. In this study, we show that aloperine can inhibit cell growth in human anaplastic thyroid cancers and multidrug-resistant papillary thyroid cancers. Moreover, it could suppress in vitro tumorigenesis and promote cellular apoptosis. Further analysis demonstrated the involvement of caspase-dependent apoptosis, including intrinsic and/or extrinsic pathways, in aloperine-induced cellular apoptosis. However, cell cycle regulation was not detected with aloperine treatment. This study suggests the potential therapeutic use of aloperine in human anaplastic thyroid cancers and multidrug-resistant papillary thyroid cancers.

## 1. Introduction

Thyroid cancer is one of the most common endocrine malignancies with the incidence rate being three times higher in women than in men [1]. However, the rates of mortality are equivalent between men and women [1]. The current clinical treatment guidelines recommend surgery and radioactive iodine therapy for thyroid cancers. There are three types of human thyroid cancers: differentiated thyroid cancer (DTC), poorly differentiated thyroid cancer (PDTC), and anaplastic thyroid cancer (ATC). Well-differentiated papillary thyroid carcinoma occurs in 80% of patients and the recurrence rate is <1% at 15 years for patients with limited surgery [1]. ATC and PDTC are rare among human thyroid cancers; however, they are aggressive, exhibit a poor prognosis, and fail to respond to the available chemotherapeutic agents and radiotherapy. Although surgical treatment and radioactive iodine therapy have been found to be effective for most patients with thyroid cancer, there are no effective treatments for patients with ATCs, PDTCs, or DTCs that have reached an advanced or recurrent level. Therefore, there is an urgent need for a novel and an effective treatment strategy or an agent for treating these cancers.

*Sophora alopecuroides* is a traditional Chinese herb that has been used for its antipyretic, anti-inflammatory, and analgesic properties [2]. Aloperine is a quinolizidine alkaloid that has been isolated from *S. alopecuroides* extract [3]. It has been reported to possess multiple pharmacological activities, including antimicrobial, antiviral, anti-allergic, anti-renal injury, anti-neuronal injury, anti-inflammatory, antinociceptive, and antitumor effects [2,3,4,5,6,7,8,9,10,11,12,13]. The antitumor effects of aloperine have also been demonstrated on several human cancer cells, including colon cancer, lung cancer, and multiple myeloma [2,8,13]; however, there is a lack of studies on its antitumor effects on human thyroid cancers and the associated mechanisms.

In this study, we investigated the in vitro antitumor effect of aloperine on various types of human thyroid cancer cells. We found that aloperine could inhibit cellular proliferation and also reduce the tumorigenesis of cells of multidrug-resistant human papillary thyroid carcinoma and in ATC. Further analysis demonstrated that intrinsic and/or extrinsic caspase-dependent apoptosis was elevated and the Akt signaling pathway was involved in aloperine-mediated cellular apoptosis. Our results suggest that aloperine could be used to develop a potential therapeutic agent for complicated human thyroid cancers.

## 2. Results

### 2.1. Aloperine Inhibits Cellular Growth and In Vitro Tumorigenesis of Human Thyroid Cancer Cells

**Figure 1:**
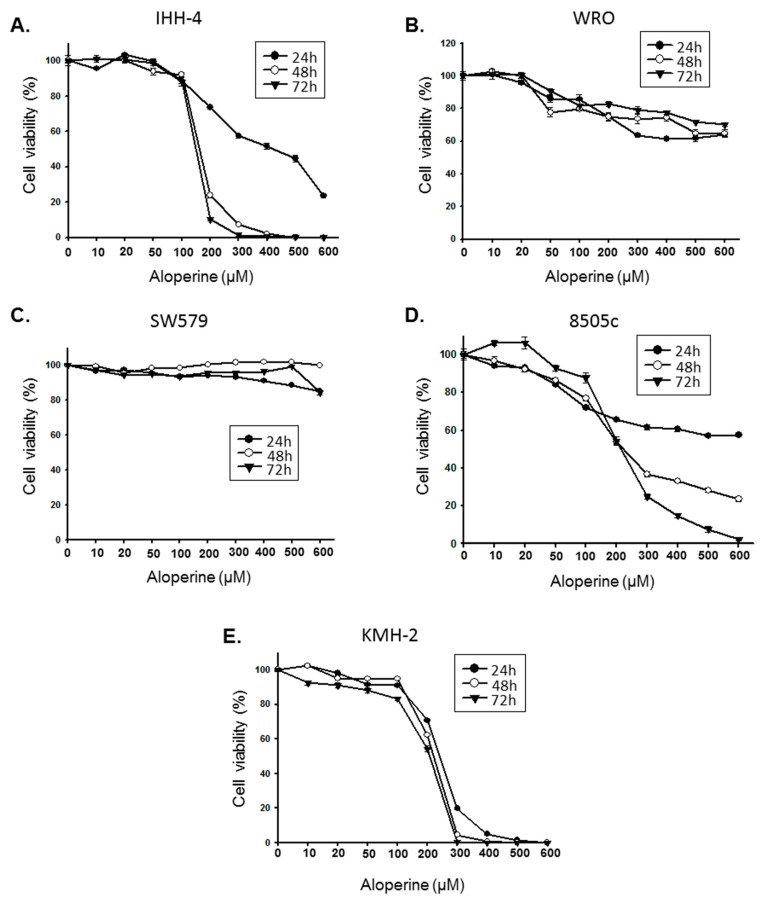
Aloperine suppressed the growth of human thyroid cancer cells. Human thyroid cancer cells, namely (**A**) IHH-4, (**B**) WRO, (**C**) SW579, (**D**) 8505c, and (**E**) KMH-2 cells were incubated with various aloperine doses, and the cellular viabilities examined 24, 48, and 72 h post-treatment using CCK-8 analysis. Dimethyl sulfoxide (DMSO) treatment was used as a negative control, and all of the groups were normalized to the control group. The results are expressed as mean ± SD of three independent experiments.

**Figure 2:**
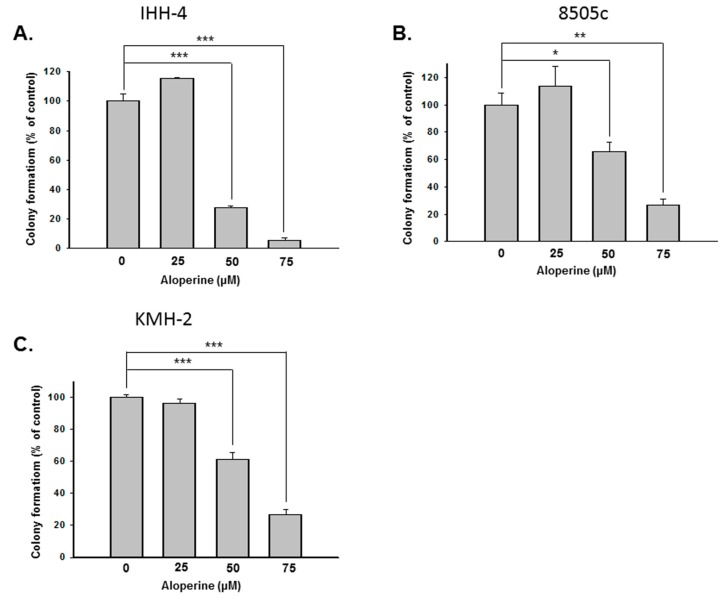
Aloperine inhibited colony formation in human thyroid cancer cells. Human thyroid cancer cell lines (**A**) IHH-4, (**B**) 8505c, and (**C**) KMH-2 were treated with aloperine and the tumorigenic activity of the cells examined by in vitro colony formation assay. The data represent mean ± SD of three separate experiments. DMSO was used as a negative control. * *p* < 0.05, ** *p* < 0.01 , *** *p* < 0.001.

To evaluate the antitumor bio-activity of aloperine on human thyroid cancer, five human thyroid cancer cell lines—including papillary thyroid carcinoma (IHH-4), follicular thyroid carcinoma (WRO), poorly differentiated thyroid carcinoma (SW579), and anaplastic thyroid carcinoma (8505c and KMH-2)—were incubated with aloperine and their cellular viabilities were examined by CCK-8 analysis. Figure 1 shows that aloperine could suppress cellular proliferation in all of the cells in a dose-dependent manner. The IHH-4, 8505c, and KMH-2 cells displayed more sensitivity to treatment with aloperine (Figure 1). The IC_50_ values of the IHH-4 cells were 423.2, 161.7, and 148.8 µM (Figure 1A); those of the 8505c cells were 708.8, 222.0, and 214.4 µM (Figure 1D); and those of the KMH-2 cells were 240.8, 221.2, and 208.0 µM (Figure 1E). Because the IHH-4, 8505c, and KMH-2 cells showed more sensitization with aloperine treatment, we further evaluated whether aloperine could suppress tumorigenesis in these cells in vitro by using a colony formation assay. Figure 2 shows that aloperine treatment suppressed colony formation in IHH-4, 8505c, and KMH-2 cells in a dose-dependent manner, suggesting bioactive tumorigenesis inhibition by aloperine on human thyroid cancers. Among these cells, IHH-4 was the most sensitive to aloperine-mediated antitumorigenesis (Figure 2).

### 2.2. Aloperine Has No Impact on Cell Cycle in Human Thyroid Cancers

**Figure 3:**
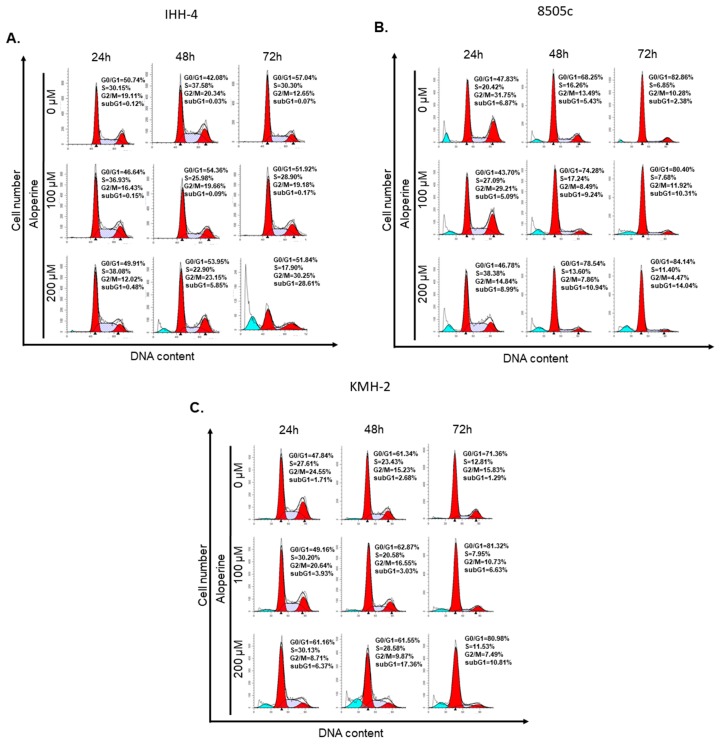
Cell cycle regulation of human thyroid cancer cells after aloperine treatment. (**A**) IHH-4, (**B**) 8505c, and (**C**) KMH-2 cells were incubated with aloperine for 24, 48, and 72 h, and the cell cycle of the cells was determined by flow cytometry after PI staining. DMSO was used as a negative control.

As aloperine has an antiproliferative effect on IHH-4, 8505c, and KMH-2 cells, we further evaluated whether it could influence the cell cycle of these cells. The cells were incubated with aloperine and the cell cycle was determined using flow cytometry analysis. Cells incubated with dimethyl sulfoxide (DMSO) were used as a negative control. We found that the cell cycle of IHH-4, 8505c, and KMH-2 cells under aloperine treatment displayed no significant cell cycle arrest compared to cells incubated with DMSO (Figure 3). These data suggest that aloperine does not regulate cell cycle to achieve its antitumor behavior. However, a significant sub-G1 group was determined in IHH-4, 8505c, and KMH-2 cells under aloperine treatment (Figure 3 and Figure S1), suggesting that cell death might happen in these cells after aloperine treatment. In addition, the G2/M population decreased in cells treated with aloperine, which might illustrate that it causes by cell death.

### 2.3. Aloperine Induces Caspase-Dependent Apoptosis in Human Thyroid Cancers

**Figure 4:**
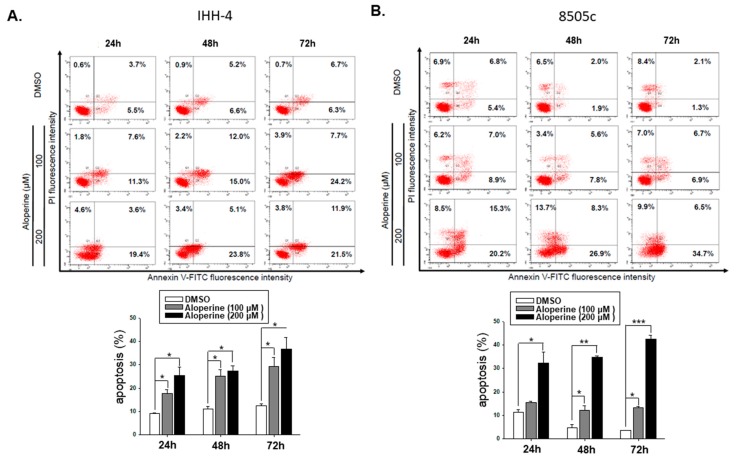

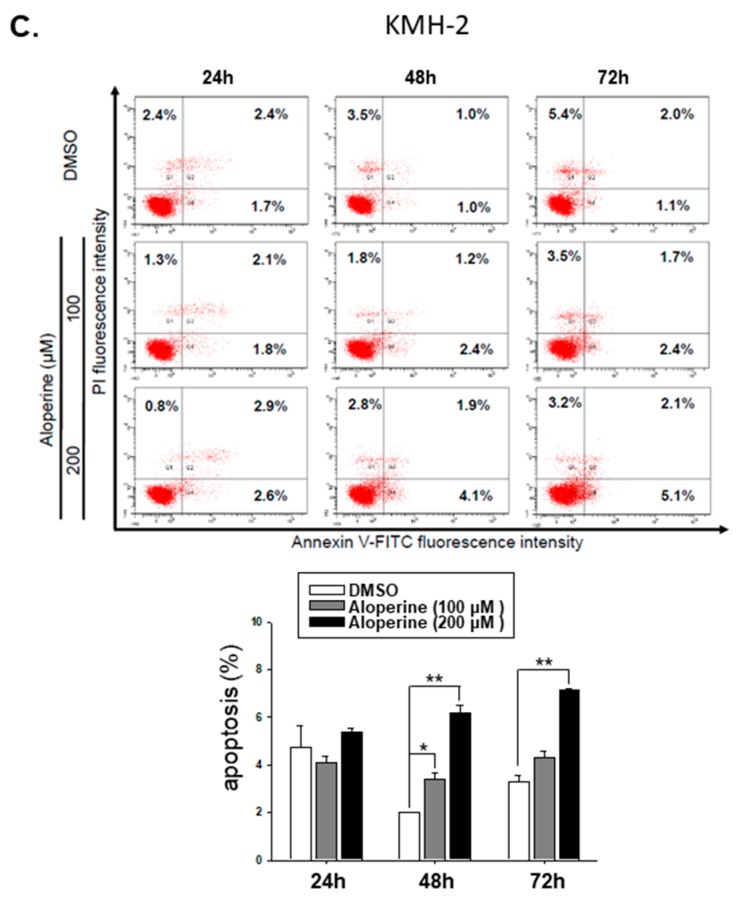
Aloperine-induced cellular apoptosis in human thyroid cancer cells. (**A**) IHH-4, (**B**) KMH-2, and (**C**) 8505c cells were incubated with aloperine for 48 h, and cellular apoptosis was detected using flow cytometry with PI/Annexin-V double staining. The results are expressed as mean ± SD of three independent experiments. DMSO was used as a negative control. * *p* < 0.05, ** *p* < 0.01 , *** *p* < 0.001.

**Figure 5:**
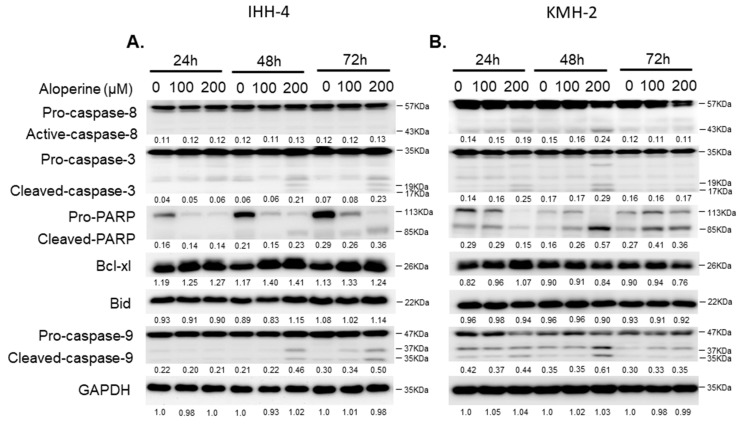

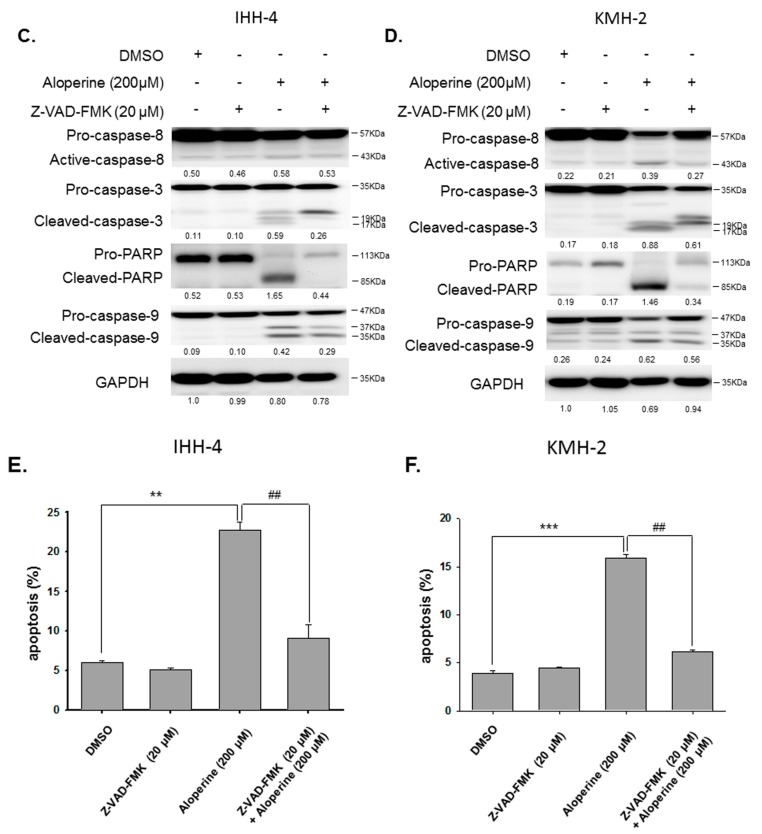
Aloperine induced caspase-dependent apoptosis in human thyroid cancer cells. (**A**) IHH-4 and (**B**) KMH-2 cells were incubated with aloperine, and the activation of caspase-8, -9 and -3 and PARP was detected by Western blot. (**C**) IHH-4 and (**D**) KMH-2 cells were pre-incubated with the pan-caspase inhibitor (Z-VAD-FMK) for 2 h and/or incubated with aloperine to confirm the activation of caspase-dependent apoptosis. The cellular apoptosis (**C,D**) was determined by flow cytometry with PI/Annexin-V double staining (**E**,**F**). Three independent experiments were confirmed. DMSO was used as a negative control. ** *p* < 0.01, *** *p* < 0.001, ^##^ *p* < 0.01.

To confirm whether aloperine mediated cellular apoptosis in human thyroid cancers, IHH-4, 8505c, and KMH-2 cells were incubated with aloperine, and the apoptotic cells were determined using flow cytometry. The data show that aloperine could induce cellular apoptosis in human thyroid cancer cells in a dose- and time-dependent manner (Figure 4). IHH-4 and KMH-2 cells showed more sensitivity toward aloperine-mediated apoptosis (Figure 4A,B). The intrinsic and extrinsic pathways of caspase-dependent apoptosis were evaluated in IHH-4 and KMH-2 cells. Figure 5A,B show that caspase-3 and poly ADP ribose polymerase (PARP) were activated under aloperine treatment in a dose- and time-dependent manner. Moreover, cleaved caspase-9 was also found in both IHH-4 and KMH-2 cells treated with aloperine, suggesting that an intrinsic pathway was elevated after aloperine treatment (Figure 5A,B). However, caspase-8 activation was only found in KMH-2 cells after incubation with aloperine, suggesting that an extrinsic pathway was also activated under aloperine treatment (Figure 5A). Furthermore, there were no significant differences in the expressions of B-cell lymphoma-extra large (Bcl-xL) and Bid in both KMH-2 and IHH-4 cells incubated with aloperine, suggesting that there was no interaction between the extrinsic and intrinsic pathways after aloperine treatment (Figure 5A,B). Moreover, Z-VAD-FMK, a pan-caspase inhibitor, was used to block the activation of caspases in IHH-4 and KMH-2 cells co-incubated with aloperine; the activation of caspase-3 and PARP were examined using Western blotting. The data show that aloperine-mediated caspase-8 and -9 activations were partially suppressed and caspase-3 activation was reduced significantly under Z-VAD-FMK treatment in IHH-4 and KMH-2 cells (Figure 5C,D). The activation of PARP was also suppressed in cells treated with Z-VAD-FMK, suggesting that blocking the activation of caspases could reduce aloperine-mediated cellular apoptosis (Figure 5C,D). Therefore, aloperine-mediated cellular apoptosis in IHH-4 and KMH-2 cells were also confirmed using flow cytometry analysis in cells co-incubated with Z-VAD-FMK. We demonstrated that aloperine-mediated cellular apoptosis was significantly reduced in cells co-incubated with Z-VAD-FMK (Figure 5E,F). The data suggest that aloperine could suppress cell growth through caspase-dependent apoptosis. In summary, in the present study, we demonstrated that aloperine could be a candidate therapeutic agent for human anaplastic and multidrug-resistant papillary thyroid carcinoma.

### 2.4. Aloperine Modulates Cellular Apoptosis through the Akt Signaling Pathway

**Figure 6:**
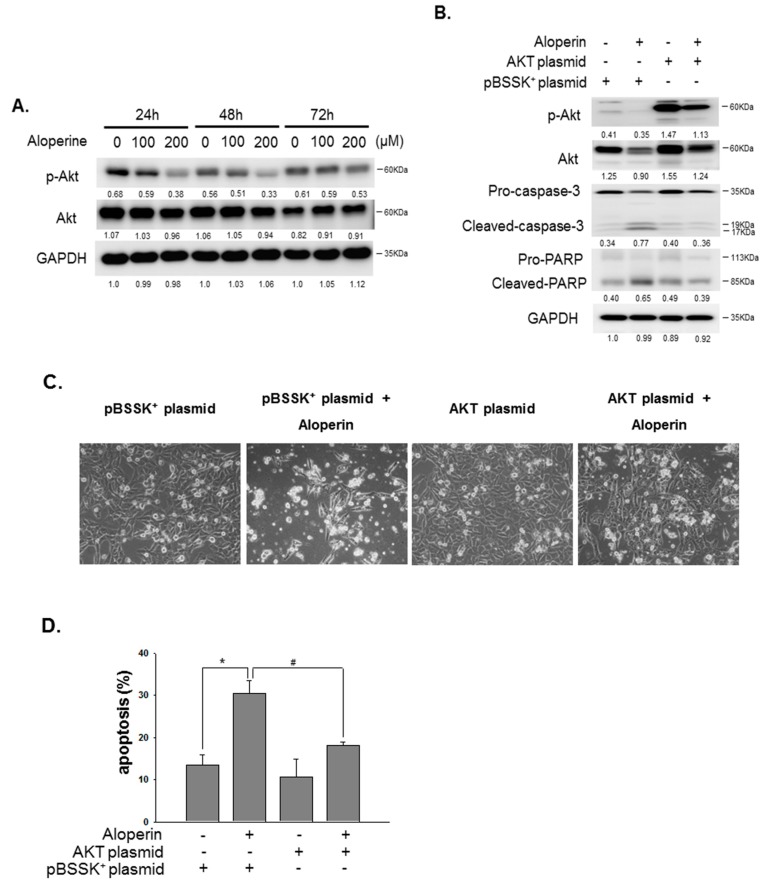
Akt signaling pathway is involved in aloperine-mediated cellular apoptosis. (**A**) KMH-2 cells were incubated with aloperine and the expressions of Akt and phospho-Akt were determined using Western blot. (**B**) To confirm the impact of the Akt pathway regulation on aloperine-mediated cellular apoptosis, cells were transiently transfected with a constitutively active Akt plasmid, and the apoptotic markers were examined using Western blot after aloperine treatment. (**C**) The cellular phenomena of the cells in (**B**) were determined by microscopy (200×). (**D**) The apoptosis of the cells in (**B**) was also confirmed by flow cytometry with PI/Annexin-V double staining. pBSSK^+^ plasmid was used as a negative control. * *p* < 0.05, ^#^ *p* < 0.05.

Phosphoinositide 3-kinase (PI3K) and the related downstream Akt pathway has been demonstrated to play an essential role in cell proliferation and in the protection of cellular apoptosis [14]. In addition, the present study shows that aloperine downregulates the activation of the Akt pathway to further induce cellular apoptosis [8]. Therefore, the expression and activation of the Akt pathway were examined in the human thyroid cancer cells under aloperine treatment. We demonstrated that cells incubated with aloperine could suppress the activation of the Akt pathway in a dose-dependent manner (Figure 6A). Aloperine partially reduced the expression of the total form of Akt in KMH-2 cells (Figure 6A) but it had no impact on IHH-4 cells. To confirm the role of aloperine modulation on the Akt pathway and cellular apoptosis, a constitutively active Akt construct was transiently transfected into KMH-2 cells and the expression of phospho-Akt was examined (Figure 6B). We have demonstrated that elevated Akt activation reduced aloperine-mediated caspase activation and PARP activation (Figure 6B). The cellular phenomena and apoptotic cells were elevated in the cells treated with aloperine; however, they were significantly reduced under activation of the Akt pathway (Figure 6C,D). These data suggest that downregulation of the Akt pathway is involved in the aloperine-mediated apoptosis in human thyroid cancer.

## 3. Discussion

We investigated the anticancer activity of aloperine in various human thyroid carcinoma cell lines. We observed that aloperine exerted effective growth inhibition activity, especially on ATC cells (8505c and KMH-2 cells) and multidrug-resistant papillary thyroid carcinoma cells (IHH-4 cells) (Figure 1). Moreover, analysis of in vitro colony formation revealed that aloperine can suppress tumorigenesis in human thyroid cancer cells (Figure 2). Previous research has shown that aloperine exerts antitumor behavior in colon cancer, lung cancer, and multiple myeloma [2,8,13]. To our knowledge, this is the first study to demonstrate that aloperine could be used as a potential and effective therapeutic agent for aggressive human thyroid carcinomas.

The previous study demonstrated that aloperine induced a G2/M-phase cell cycle arrest, and inhibited Janus kinase (JAK)/Signal transducer and activator of transcription 3 (Stat3), phosphatidylinositol 3-kinase/Akt, as well as Bcl-2, in a human colon cancer cell line [2]. However, in the present study, regulation of the cell cycle in the aloperine-treated human thyroid cancer cells was similar to that in the DMSO-treated group (Figure 3). This discrepancy in the results might be due to genetic variations in the cancer cell types. In addition, we have demonstrated that the Akt pathway is involved in aloperine-mediated caspase-dependent apoptosis. Based on Zhang et al.’s report [2], we suggest that aloperine might suppress the activation of Akt pathway and the downstream Bcl-2 expression to modulate cellular apoptosis in human thyroid cancers.

In addition, aloperine induced apoptosis in the IHH-4, 8505c, and KMH-2 cells in a dose-dependent manner (Figure 4) and blocking the aloperine-mediated apoptosis could partially reverse the antitumor behavior of aloperine in human thyroid cancers (Figure 5E,F). These results suggest that aloperine exerts its antitumor activity through the elevation of cellular apoptosis. However, we cannot exclude the possible involvement of caspase-independent apoptosis or autophagy in the aloperine-mediated effects on human thyroid cancers. This finding is consistent with previous reports on human leukemia, multiple myeloma, and colon cancers [2,5,8]. We also demonstrated that a caspase-dependent apoptosis was induced after aloperine treatment in KMH-2 and IHH-4 cells (Figure 5). Furthermore, the intrinsic pathway was elevated in the aloperine-treated IHH-4 cells, whereas both the intrinsic and extrinsic pathways were activated in the aloperine-treated KMH-2 cells (Figure 5A,B). This difference might be due to the genetic variation of these cancer cells, and further investigation is needed to identify the mechanisms in more detail.

The PI3K/Akt signaling pathway has been shown to play a central role in cellular proliferation, migration, invasion, and apoptosis in various human cancers [14]. The overexpression of the PI3K/Akt pathway appears to be related to malignancies and poor prognosis in multiple human cancers [14]. In addition, the PI3K/Akt pathway has been demonstrated to be a feature of follicular carcinomas and ATCs, but it is less frequent in papillary thyroid carcinoma [15]. Genetic mutation, changes in gene copy number, and protein expression and stability may contribute to the overactivation of the PI3K/Akt pathway in the former cases [15]. Moreover, targeting the PI3K/Akt pathway has been demonstrated to be an effective therapeutic strategy for treating both human solid cancers and thyroid carcinomas [16,17]. In the previous study, aloperine has been shown to suppress phosphorylation of the phosphatase and tensin homolog (PTEN) and Akt pathway and further elevate caspase-9-related cellular apoptosis [8]. In agreement with this finding, we also found caspase-dependent apoptosis (Figure 5) and suppression of Akt activation in aloperine-treated IHH-4 and KMH-2 cells (Figure 6). Moreover, we further demonstrated that aloperine displayed an anti-human thyroid carcinoma behavior through the modulation of Akt-related cellular apoptosis (Figure 6). Loss of PTEN is frequently detected with progression to malignancy and an aggressive phenotype in human thyroid carcinomas [15]. Further investigation of the regulation of PTEN and aloperine-mediated apoptosis in human thyroid cancers is needed. Importantly, no differences were detected in the levels of creatinine, hemoglobin, and bilirubin in vivo in aloperine-treated mice, suggesting that aloperine is safe and nontoxic in vivo [8]. Therefore, to our knowledge, this is the first study to demonstrate that aloperine can inhibit tumor cell growth and tumorigenesis and promote the induction of apoptosis in various human thyroid cancers, especially in multidrug-resistant papillary thyroid carcinoma and ATC. However, in vivo studies are needed to analyze the safety and efficacy of aloperine in treating human thyroid carcinomas.

## 4. Materials and Methods

### 4.1. Cell Line Culture

Human thyroid cancer cell lines including IHH-4 (papillary thyroid carcinoma), WRO (follicular thyroid carcinoma), SW579 (poorly differentiated thyroid carcinoma), 8505c (anaplastic thyroid carcinoma), and KMH-2 (anaplastic thyroid carcinoma) were purchased from BCRC (Bioresource Collection and Research Center, Hsinchu, Taiwan) and JCRB (Japan Collection of Research Bioresources Cell Bank, Tokyo, Japan). Among them, IHH-4 and KMH-2 cells were cultured with DMEM + RPMI (1:1) medium (GIBCO, Gaithersburg, MD, USA), WRO cells were cultured with RPMI medium (GIBCO), SW579 cells were cultured with L15 medium (GIBCO), and 8505c cells were cultured with MEM medium (GIBCO) supplemented with 10% FBS (Biological Industries, Kibbutz Beit Haemek, Israel) at 37 °C in a 5% CO_2_ incubator.

### 4.2. Cell. Viability Assay

Cells (5 × 10^3^ cells/well) were seeded in 96-well cell culture plates and maintained with the aforementioned medium. After an overnight incubation, the attached cells were incubated with control medium (containing 0.01% dimethyl sulfoxide, DMSO) or aloperine-containing medium (Selleck Chemicals, Houston, TX, USA). The cellular viability was examined by the CCK-8 assay kit (Enzo Life Sciences, Farmingdale, NY, USA) after treatment. Three independent experiments were performed. 

### 4.3. Colony Formation Assay

The colony formation assay has been described previously [18]. Briefly, cells (10^3^ cells/well) were plated in 6-well plates. Cells were exposed to various doses of drug or DMSO as a negative control for 12 days. Cells were then fixed and stained with 10% crystal violet (Sigma-Aldrich, St. Louis, MO, USA), and the colony forming efficiency were determined and counted in three independent experiments.

### 4.4. Cell. Cycle Analysis

The cell cycle analysis has been described previously [19,20,21]. Cells (10^6^ cells/dish) were seeded in 10-cm cell culture dish for overnight. The attached cells were incubated with aloperine or DMSO for various durations. The cell cycles of these cells were determined by flow cytometry analysis (BD Biosciences, San Jose, CA, USA) after PI staining (Sigma-Aldrich), and the distribution of the cell cycles were identified by Modfit LT software (BD Biosciences, New York, NY, USA).

### 4.5. Cellular Apoptosis Analysis

Cells were treated with DMSO or aloperine for various durations. The procedure for determining apoptosis was as previously reported [19,20,21]. First, cells treated with DMSO or aloperine were stained with PI/Annexin-V (Sigma-Aldrich) and the apoptotic cells were determined by flow cytometry. To determine the mechanisms involved in aloperine-mediated apoptosis, the expressions of PARP, Bcl-xL, Bid, caspase-3, -8, and -9 in the cells incubated with drugs were examined by Western blot analysis. All of the primary antibodies used in this study were purchased from Cell Signaling Technology Inc. (Danvers, MA, USA). Moreover, Z-VAD-FMK (ApexBio Technology, Houston, TX, USA) was used to block and confirm the aloperine-mediated caspase-dependent apoptosis. In addition, plasmid pHRIG-Akt1 (a constitutively active human Akt1 construct purchased from Addgene) and pBSSK^+^ (as a negative control) was transfected with Lipofectamine 2000 (Thermo Scientific, Waltham, MA, USA) according to the manufacturer’s instruction.

### 4.6. Statistical Analysis

Data are presented as the mean ± standard error for the indicated number of separate experiments. Statistical differences were analyzed by one-way ANOVA and Fisher’s least significant difference test. Statistical significance was defined as *p* < 0.05 in all tests.

## 5. Conclusions

Our research reveals that aloperine is a promising candidate for the prevention and treatment of human anaplastic thyroid cancers and multidrug-resistant papillary thyroid cancers.

## Supplementary Materials

Supplementary materials can be found at www.mdpi.com/1422-0067/19/1/312/s1.

## References

[1] Siegel R.L., Miller K.D., Jemal A. (2017). Cancer Statistics. CA Cancer J. Clin..

[2] Zhang L., Zheng Y., Deng H., Liang L., Peng J. (2014). Aloperine induces G2/M phase cell cycle arrest and apoptosis in HCT116 human colon cancer cells. Int. J. Mol. Med..

[3] Zhou C.C., Gao H.B., Sun X.B., Shi H.B., Liu W., Yuan H.N., Wang Z.X. (1989). Anti-inflammatory and anti-allergic action of aloperine. Acta Pharmacol. Sin..

[4] Yuan X.Y., Liu W., Zhang P., Wang R.Y., Guo J.Y. (2010). Effects and mechanisms of aloperine on 2,4-dinitrofluorobenzene-induced allergic contact dermatitis in BALB/c mice. Eur. J. Pharmacol..

[5] Lin Z., Huang C.F., Liu X.S., Jiang J. (2011). In vitro anti-tumour activities of quinolizidine alkaloids derived from Sophora flavescens Ait. Basic Clin. Pharmacol. Toxicol..

[6] Lin W.C., Lin J.Y. (2011). Five bitter compounds display different anti-inflammatory effects through modulating cytokine secretion using mouse primary splenocytes in vitro. J. Agric. Food Chem..

[7] Ma N.T., Zhou R., Chang R.Y., Hao Y.J., Ma L., Jin S.J., Du J., Zheng J., Zhao C.J., Niu Y. (2015). Protective effects of aloperine on neonatal rat primary cultured hippocampal neurons injured by oxygen-glucose deprivation and reperfusion. J. Nat. Med..

[8] Wang H., Yang S., Zhou H., Sun M., Du L., Wei M., Luo M., Huang J., Deng H., Feng Y. (2015). Aloperine executes antitumor effects against multiple myeloma through dual apoptotic mechanisms. J. Hematol. Oncol..

[9] Hu S., Zhang Y., Zhang M., Guo Y., Yang P., Zhang S., Simsekyilmaz S., Xu J.F., Li J., Xiang X. (2015). Aloperine protects mice against ischemia reperfusion (IR)-induced renal injury by regulating PI3K/AKT/mTOR signaling and AP-1 activity. Mol. Med..

[10] Dang Z., Zhu L., Lai W., Bogerd H., Lee K.H., Huang L., Chen C.H. (2016). Aloperine and Its Derivatives as a New Class of HIV-1 Entry Inhibitors. ACS Med. Chem. Lett..

[11] Ren D., Ma W., Guo B., Wang S. (2017). Aloperine attenuates hydrogen peroxide-induced injury via anti-apoptotic activity and suppression of the nuclear factor-κB signaling pathway. Exp. Ther. Med..

[12] Wu F., Hao Y., Yang J., Yao W., Xu Y., Yan L., Niu Y., Sun T., Yu J., Zhou R. (2017). Protective effects of aloperine on monocrotaline-induced pulmonary hypertension in rats. Biomed. Pharmacother. Biomed. Pharmacother..

[13] Xu Z., Yan Y., Zeng S., Qian L., Dai S., Xiao L., Wang L., Yang X., Xiao Y., Gong Z. (2017). Reducing autophagy and inducing G1 phase arrest by aloperine enhances radio-sensitivity in lung cancer cells. Oncol. Rep..

[14] Franke T.F., Hornik C.P., Segev L., Shostak G.A., Sugimoto C. (2003). PI3K/Akt and apoptosis: Size matters. Oncogene.

[15] Robbins H.L., Hague A. (2015). The PI3K/Akt Pathway in Tumors of Endocrine Tissues. Front. Endocrinol..

[16] Pons-Tostivint E., Thibault B., Guillermet-Guibert J. (2017). Targeting PI3K Signaling in Combination Cancer Therapy. Trends Cancer.

[17] Yi H., Ye X., Long B., Ye T., Zhang L., Yan F., Yang Y., Li L. (2017). Inhibition of the AKT/mTOR Pathway Augments the Anticancer Effects of Sorafenib in Thyroid Cancer. Cancer Biother. Radiopharm..

[18] Lu C.H., Chen S.H., Chang Y.S., Liu Y.W., Wu J.Y., Lim Y.P., Yu H.I., Lee Y.R. (2017). Honokiol, a potential therapeutic agent, induces cell cycle arrest and program cell death in vitro and in vivo in human thyroid cancer cells. Pharmacol. Res..

[19] Hua S.C., Chang T.C., Chen H.R., Lu C.H., Liu Y.W., Chen S.H., Yu H.I., Chang Y.P., Lee Y.R. (2012). Reversine, a 2,6-disubstituted purine, as an anti-cancer agent in differentiated and undifferentiated thyroid cancer cells. Pharm. Res..

[20] Lu Y.C., Lee Y.R., Liao J.D., Lin C.Y., Chen Y.Y., Chen P.T., Tseng Y.S. (2016). Reversine Induced Multinucleated Cells, Cell Apoptosis and Autophagy in Human Non-Small Cell Lung Cancer Cells. PLoS ONE.

[21] Shi C.S., Li J.M., Chin C.C., Kuo Y.H., Lee Y.R., Huang Y.C. (2017). Evodiamine Induces Cell Growth Arrest, Apoptosis and Suppresses Tumorigenesis in Human Urothelial Cell Carcinoma Cells. Anticancer Res..

