# Supplementary material for: In Vitro Antitumor Activity of Aloperine on Human Thyroid Cancer Cells through Caspase-Dependent Apoptosis
Source: Int J Mol Sci. 2018 Jan 21;19(1):312. doi: 10.3390/ijms19010312 (PMC5796256; doi:10.3390/ijms19010312)
Supplement: Supplementary file 1 [file ijms-19-00312-s001.pdf]

In Vitro Antitumor Activity of Aloperine on Human Thyroid Cancer Cells through Caspase-Dependent Apoptosis

Ying-Ray Lee, Shu-Hsin Chen, Ching-Yen Lin, Wen-Ying Chao, Yun-Ping Lim, Hui-I Yu, and Chieh-Hsiang Lu

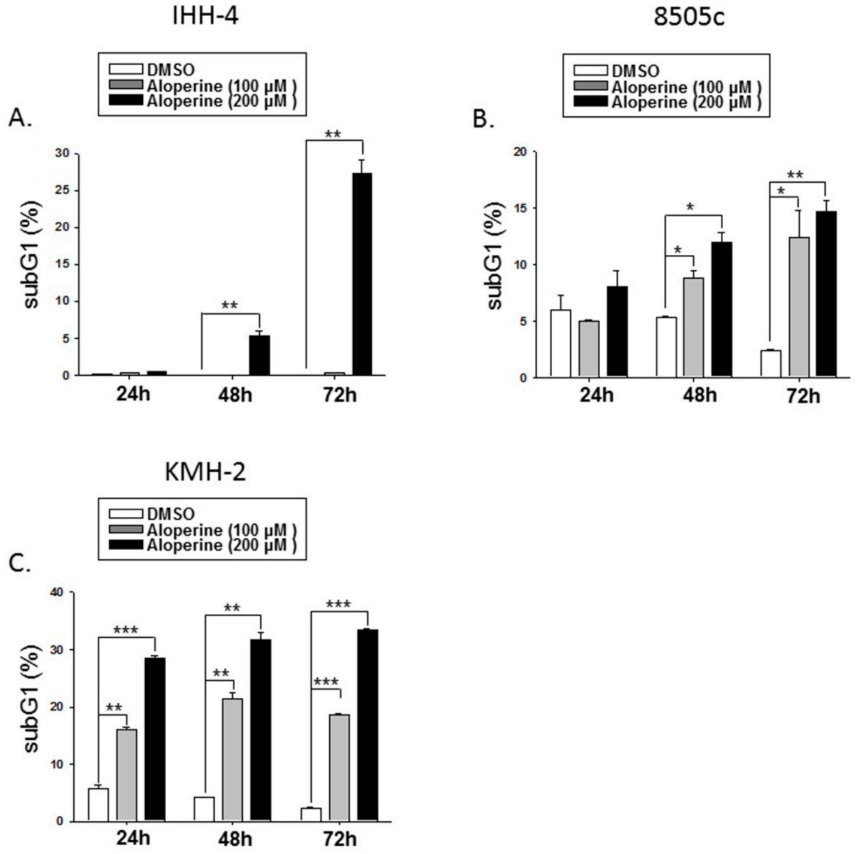

Figure S1. Sub-G1 induction of human thyroid cancer cells after aloperine treatment. (A)IHH-4 (B) 8505c and (C) KMH-2 cells were incubated with aloperine for 24, 48 and 72 h, and the cell cycle were determined by flow cytometry after PI staining. The sub-G1 population was determined and quantified. The data represent mean ± SD of three separated experiments. DMSO was used as a negative control. * $p < 0.05$, ** $p < 0.01$, *** $p < 0.001$.
